# Natural History of NAFLD/NASH

**DOI:** 10.1007/s11901-017-0378-2

**Published:** 2017-11-13

**Authors:** Mattias Ekstedt, Patrik Nasr, Stergios Kechagias

**Affiliations:** Department of Gastroenterology and Hepatology, Department of Medical and Health Sciences, Linköping University, Linköping, Sweden

**Keywords:** Natural history, Non-alcoholic fatty liver disease, Non-alcoholic steatohepatitis, Prognosis, Mortality, Liver histology

## Abstract

**Purpose of Review:**

The purpose of this review is to summarize the latest knowledge on the natural history of non-alcoholic fatty liver disease (NAFLD). The review focuses on mortality, liver-related complications, and histological course.

**Recent Findings:**

Studies during the last decade have established NAFLD as a potentially progressive liver disease. Age and diabetes are the strongest clinical predictors of progressive disease. Fibrosis stage is the most important histological variable to predict mortality and liver-related complications. So far, no study has been able to show that non-alcoholic steatohepatitis at baseline predicts mortality or future liver-related complications when adjusting for fibrosis.

**Summary:**

The outlines of the natural history of NAFLD have become clearer during the last decade. There is limited data on factors that predict clinical progression. Prospective longitudinal studies are needed to help us predict worse outcome in individual patients.

## Introduction

Non-alcoholic fatty liver disease (NAFLD) is the most common chronic liver disease with a worldwide prevalence of 20–30% [1]. Obesity entails a 3.5-fold increased risk of developing NAFLD [2], and the prevalence of NAFLD therefore mirrors the steady increase of obesity globally [3, 4]. NAFLD is strongly associated with the metabolic syndrome and type 2 diabetes mellitus (T2DM) [5•, 6]. The relationship between NAFLD and T2DM is bidirectional with a higher prevalence of NAFLD in patients with T2DM and an increased incidence of T2DM in patients with NAFLD [7–9]. The potentially progressive nature of the disease is well established, and NAFLD will become the leading cause for liver transplantation in the next few years [10].

NAFLD encompasses a histological spectrum ranging from isolated steatosis, i.e., non-alcoholic fatty liver (NAFL), to non-alcoholic steatohepatitis (NASH), cirrhosis, and hepatocellular carcinoma (HCC). NASH is characterized by hepatocellular accumulation of fat accompanied by lobular inflammation and ballooning of hepatocytes. Until recently, NAFL has been considered a benign disease and NASH the progressive disease state with risk of development of liver-related complications and increased mortality from hepatic and non-hepatic causes.

Steatosis grade and fibrosis stage can be assessed by non-invasive techniques. Ultrasonography has excellent sensitivity and specificity for moderate and severe steatosis but is less sensitive for lower grades of fatty infiltration. Fibrosis stage can be assessed non-invasively with shear wave and transient elastography [11]. Magnetic resonance imaging (MRI) can with great accuracy assess the amount of steatosis and magnetic resonance elastography (MRE) looks very promising in assessment of fibrosis [11, 12•]. On the other hand, the necroinflammatory changes, i.e., lobular inflammation and ballooning which are the hallmark of steatohepatitis, require a liver biopsy for reliable diagnosis.

The natural history of NAFLD is still somewhat unclear. Older studies reported limited numbers of highly selected patients, i.e., patients with histologically proven NAFLD who had been referred to specialized tertiary care centers with short and/or highly variable follow-up time [13–18]. There was, and still is, a shortage of population-based studies to determine the long-term prognosis of NAFLD, and it remained uncertain whether previously reported morbidity rates could be generalized to community-based practice where patients may have a milder disease. This review will focus on studies investigating the natural history of NAFLD with focus on mortality, end-stage liver disease, and histopathological course.

## Mortality

Mortality in NAFLD patients has been investigated in a number of studies during the last two decades. Increased mortality has been shown in several studies comparing NAFLD patients with a reference population [19–21, 22••]. The main cause of death in follow-up studies with biopsy-proven NAFLD is cardiovascular disease (HR 1.55–1.85), and liver-related complications are usually the third leading cause of death, with a marked increase in risk (HR 3.2–6.5) [22••, 23••]. The increased risk of all-cause mortality in NAFLD patients was confirmed in a meta-analysis (OR 1.40, 95% CI 1.23–1.60, *p* < 0.00001) [24]. However, in a newly published meta-analysis, the increased mortality of NAFLD patients, whereof the majority were diagnosed with ultrasonography, could not be confirmed (HR 1.14, 95% CI 0.99–1.32) [25•].

The relationship between NAFLD and cardiovascular disease (CVD) has been somewhat unclear. In a systematic review and meta-analysis including 34 studies, no increase in CVD mortality (HR 1.10, 95% CI 0.86–1.41) was observed [25•]. Nevertheless, NAFLD was associated with an increased risk of prevalent and incident CVD (OR 1.81 and HR 1.37, respectively). A contemporary meta-analysis showed similar results without any overall increase in CVD mortality in NAFLD, although an increased risk was seen in patients with severe NAFLD (OR 3.28, 95% CI 2.26–4.77) [26•]. In a 10-year prospective study of NAFLD patients, the risk of developing major cardiovascular events was higher in patients with NAFLD than that in controls [27]. Albeit this was partially explained by the higher presence of plaques and increased carotid intima media thickness, interestingly, hepatic steatosis was an independent predictor. Moreover, in a recent 5-year prospective cohort study, 612 patients undergoing coronary angiography were screened for presence of ultrasonographic signs of hepatic steatosis, and the majority (58.2%) was diagnosed with NAFLD [28]. Coronary artery stenosis > 50% was more frequent in NAFLD patients (84.6 vs. 64.1%, *p* < 0.001) and the need of percutaneous coronary intervention more common (68.3 vs. 43.4%, *p* < 0.001). Nonetheless, NAFLD patients had a lower risk of cardiovascular death (*p* = 0.006) and similar risk of cardiovascular events (*p* = 0.054) compared to the control group.

Previously published studies have investigated which clinical, biochemical, and histological variables that predict overall mortality in NAFLD patients. The most significant clinical predictors are age and T2DM [19, 23••, 29]. Smoking was, in a univariate analysis, associated with mortality in the study by Adams et al. [19]. The biochemical predictors best predicting long-term outcomes are fibrosis scores based on, either solely, laboratory tests, or a mixture of biochemical and clinical data [30].

The association between NASH and mortality is not completely clear. In the meta-analysis by Musso et al., NASH was associated with higher risk of all-cause and disease-specific mortality compared to isolated steatosis [24]. This relationship was also demonstrated in the study by Ekstedt et al. from 2006, included in the meta-analyses, in which NASH was associated with increased mortality [20]. In this context, it is important to remember that the vast majority of NASH patients in the Ekstedt study were classified as NASH due to presence of fibrosis at the histopathological evaluation.

In recent years, long-term follow-up studies with biopsy-proven NAFLD have shown that only liver fibrosis stage at baseline, and no other histological features, predict all-cause and disease-specific mortality [22••, 23••, 31]. In a meta-analysis by Dulai et al., liver fibrosis showed an exponentially increasing effect of all-cause mortality for each stage of fibrosis, although the study did not include age at biopsy as a cofactor [32•].

## Liver-Related Complications

Using the resources of the Rochester Epidemiology Project, Adams et al. conducted a population-based cohort study to examine the natural history of patients diagnosed with NAFLD in Olmsted County, Minnesota [19]. During a mean follow-up of 7.6 (4.0) years, 13 (3.1%) patients developed cirrhosis-related complications, including ascites in 2%, jaundice in 2%, portosystemic encephalopathy in 2%, variceal bleeding in 1%, and HCC in 0.5%. The majority (348 out of 420) of subjects in the study by Adams et al. was diagnosed with imaging.

Ekstedt et al. studied 129 subjects with biopsy-proven NAFLD [20]. Follow-up was longer (13.7 ± 1.3 years), and all patients were followed for more than 10 years. Seven patients (5.4%) developed cirrhosis-related complications during follow-up including ascites in 4%, variceal hemorrhage in 0.8%, and HCC in 2%. Subgroup analysis showed that no patient with absence of fibrosis or stage 1 fibrosis at baseline had developed liver-related complications during follow-up. One of the 4 patients (25%) with cirrhosis at baseline, 3 of the 22 patients (14%) with stage 2 fibrosis at baseline, and 3 of the 12 patients (25%) with stage 3 fibrosis at baseline developed end-stage liver disease during follow-up.

In a longitudinal, international, multicenter cohort study, Angulo et al. evaluated an outcome in 619 patients with biopsy-proven NAFLD [23••]. Over a median follow-up period of 12.6 years, 44 patients (7%) developed liver-related events including 5 subjects (0.8%) that were diagnosed with HCC. Of the 44 patients that developed end-stage liver disease, 17 died from the complications.

NAFLD is in most cases a slowly progressive disease, and the majority of NAFLD patients will never develop end-stage liver disease. However, as shown above, a significant proportion will experience liver-related complications. Predicting which NAFLD patients that will develop liver-related complications in the future and when this will occur is extremely difficult. Recently, Hagström et al. evaluated clinical outcome in 646 patients with biopsy-proven NAFLD over a mean follow-up period of 20 years [33••]. The strongest predictor for development of future liver-related complications was fibrosis stage at baseline. The presence of NASH defined according to the FLIP algorithm [34] had no impact on future outcomes. Mean time until 10% of patients had developed severe liver disease was between 31 and 36 years in patients with absence of fibrosis or stage 1 fibrosis, 19 years in stage 2 fibrosis, and 6 years in stage 3 fibrosis. Mean time until 10% of patients had developed decompensation was 6 years in cirrhosis.

NAFLD patients with advanced fibrosis or cirrhosis should be monitored closely since the risk of liver-related complications in the near future is increased. In the study by Bhala et al., 247 NAFLD patients with fibrosis stage 3 or 4 were followed [35]. Patients had well-compensated liver disease and no overt hepatic dysfunction at presentation. Mean follow-up was 7.1 years, and their natural history was compared with 264 patients with HCV infection with fibrosis stage 3 or 4 of the same functional status and a mean follow-up time of 6.2 years. Stage 4 fibrosis, past history of coronary heart disease, lower levels of serum cholesterol, lower levels of ALT, and lower platelet count were all independently associated with total liver-related complications in the NAFLD group. In this group, there was also strong evidence of differences between fibrosis stage 3 and 4 for total liver-related complications. When adjusting for baseline differences in age and gender, the cumulative incidence of liver-related complications was lower in the NAFLD than that in the HCV cohort.

HCC has during recent years been recognized as a complication of increasing significance in NAFLD, particularly in individuals with cirrhosis [36, 37]. Of the HCC cases in the USA, 55% is related to HCV, 16% to alcoholic liver disease, 14% to NAFLD, and 9% to HBV. From 2004 to 2009, the number of NAFLD-HCC showed a 9% annual increase. NAFLD-HCC were older, had shorter survival time, more heart disease, and were more likely to die from their primary liver cancer [38•].

## Histological Course

To date, there are 13 studies reporting histological development of NAFLD using paired biopsies, with a total of 521 patients included and a median follow-up time between biopsies ranging from 3 to 13.8 years [13–15, 18, 20, 39–44, 45•, 46••]. Because the definition of NASH and fibrosis stage has changed over time, comparisons are difficult to make. A majority of the studies are retrospective with patient cohort selected through histological records with NASH dominating the histological diagnosis. In 10 studies [13, 15, 18, 20, 39, 41–44, 46••], fibrosis stage at baseline and follow-up is reported (*n* = 416) with 37% of patients progressing in fibrosis stage (153/416) and 12% progressing from low stage fibrosis (F0-F2) to advanced fibrosis (F3-F4). The number of patients developing advanced fibrosis is probably an underestimation since patients with signs of decompensation are not included to undergo a second biopsy. A recent systematic review and meta-analysis of 11 paired biopsy studies, including 411 patients and 2145.5 person years of follow-up, showed that 33.6% had fibrosis progression [47•]. The overall, annual fibrosis progression rate was found to be 0.07 stages for NAFL and 0.14 stages for NASH, corresponding to one stage of fibrosis progression over a median of 14.3 and 7.1 years, respectively.

Since isolated steatosis has been considered a benign condition, few studies have focused on assessing the natural history of patients with isolated steatosis. In a study by Teli et al., 12 patients were included and follow-up period ranged from 7.6 to 16 years [15]. Only one patient progressed to stage 1 fibrosis. In a recent British study with serial biopsies of 108 patients, 17 were diagnosed with isolated steatosis of whom 4 (24%) showed signs of fibrosis progression [46••].

Although our knowledge of isolated steatosis is limited, new studies on NAFL have recently emerged. Three recent studies have demonstrated fibrosis progression in a significant proportion of NAFL patients [44, 45•, 46••]. In a prospective study by Wong et al., 29 patients with serial liver biopsies 3 years apart were diagnosed with NAFL (defined as NAFLD activity score < 3) [44]. In that study, 28% showed fibrosis progression. In two recent studies by Pais et al. and McPherson et al., NAFL was defined as isolated steatosis or steatosis with mild inflammation (grade 1), without ballooning and fibrosis stage < 3. Fibrosis progression was observed in 16 out of 52 patients (31%). In the retrospective study by Pais et al., 6 out of 25 patients (24%) with NAFL progressed to bridging fibrosis [45•], and in the more recent study by McPherson et al., 10 out of 27 patients (37%) with NAFL showed fibrosis progression [46••]. In the meta-analysis by Singh et al. [47•], NAFL seem to portend a slower progression; however, 5 out of 29 patients with NAFL and baseline fibrosis stage 0 (17%) and 2 out of 11 patients with NASH and baseline fibrosis stage 0 (18%) developed advanced fibrosis within 6 years.

Predicting fibrosis progression using baseline biochemical, clinical, or histological parameters is difficult. Presence of NASH or NAFLD activity score (NAS) at baseline has never correlated with fibrosis progression. However, NASH or high NAS were more prevalent in the follow-up biopsies in patients with disease progression.

In a recent abstract by Sanyal et al., 477 patients with NASH and advanced fibrosis (Ishak stages 3–6) had serial liver biopsies performed after a median of 2.1 years (range, 0–3.5) [48]. Baseline NAS did not predict progressive fibrosis. Among baseline factors, Ishak stage ≥ 4 was associated with progression. These findings are in accordance with previous studies using paired biopsies in which higher baseline fibrosis stage portend a more rapid fibrosis progression. Another abstract by McPherson et al. included 321 patients with NAFLD who had sequential biopsies conducted over a median follow-up of 4.1 years (range, 1–22.6), showed fibrosis progression in 35%, with no difference in fibrosis progression based on fibrosis stage or NASH/NAFL at baseline. However, moderate to severe steatosis seemed associated with fibrosis progression [49].

## Discussion

Over the years, the definitions of the different stages of the NAFLD spectrum have varied [50]. The entity NASH was introduced in the seminal paper by Ludwig and coworkers back in 1980 due to the striking similarities between alcoholic steatohepatitis and NASH [51]. The widely used Brunt criteria were the first commonly used system to grade and stage the histopathological features of NASH [52]. It is important to point out that the Brunt paper did not provide a definition of NASH; it was based on 51 liver biopsies in which the pathologist had already diagnosed steatohepatitis. The definition of NASH was made by the pathologist, and the scoring system was designed to define the activity and the stage of the disease. A further development of the scoring system was proposed by Kleiner et al., who introduced the NAS [53]. The NAS was derived from 50 cases (32 adult cases and 18 pediatric) diagnosed as “NASH,” “borderline NASH,” or “not NASH” by eight experienced liver pathologists. The NAS was defined as the unweighted sum of steatosis (0–3), lobular inflammation (0–2), and ballooning (0–2). A NAS > 4 was statistically correlated with an independent diagnosis of NASH. Since its introduction, the NAS has been widely misused and many authors have used it to define NASH. However, the NAS system was not intended to categorize patients according to their NASH status; instead, it is meant to be used to evaluate changes in individual histological features.

More recently, an algorithm to diagnose NASH was published by Bedossa and coworkers in which steatosis, lobular inflammation, and ballooning constitute mandatory parameters [34]. A score (SAF score), including Steatosis, Activity (ballooning + lobular inflammation), and Fibrosis summarizing the main histological lesions, was proposed. Thus, there has not been a solid definition of NASH, which is important to remember when comparing follow-up studies published at different time points. Moreover, none of the scoring systems has been derived by the ability of the score to predict future clinical outcomes. Moreover, there is a strong correlation between necroinflammatory changes and fibrosis stage [31] which in combination with the slowly progressive nature of NAFLD complicates the identification of causality between histological features and future development of end-stage liver disease or risk for increased mortality.

Published studies using paired biopsies have shown that NAFLD is a progressive disease of variable rate in a significant proportion of patients. Rapid fibrosis progression has previously been thought to be driven by the presence of inflammation. Noteworthy, there is no large follow-up study [22••, 23••, 31] showing that necroinflammatory changes at baseline predict future mortality or development of end-stage liver disease. However, it cannot be ruled out that necroinflammation develops during disease progression and acts as a key player influencing the course of NAFLD.

Recent studies show that the natural histological development of NAFLD is surprisingly unpredictable. No or few baseline clinical, biochemical, and histological parameters predict future histological development. This was well demonstrated in the study by McPherson et al. [46••], although follow-up time between biopsies was short. Identical findings were confirmed in two newly presented abstracts, where baseline NAS did not predict progression of fibrosis stage, although the presence of moderate to severe steatosis was associated with fibrosis progression (*p* < 0.0001) [48, 49].

Increased mortality in NAFLD patients is not clear. Although prospective follow-up studies have shown a slight increase in mortality [19, 20, 22••, 23••], a recent meta-analysis rejected the relationship [25•]. This meta-analysis included studies which used imaging technology to diagnose NAFLD [25•]. The cut-off to detect steatosis on ultrasonography is approximately 10% [54]. However, the widely used threshold for diagnosing NAFLD with ^1^H-MRS proton density fat fraction (PDFF) is 5 or 5.56% [55]. In a recently published study [12•], in which, contrary to previous studies, gold standard liver biopsy was used to define steatosis; it was shown that the PDFF threshold for diagnosing NAFLD with ^1^H-MRS is too high and should be reduced to at least 3%. Follow-up studies have shown that as fibrosis stage increases, the grade of steatosis usually decreases. Therefore, the use of ultrasonography as a diagnostic tool for NAFLD increases the risk of excluding NAFLD patients with mild steatosis, and in some cases advanced fibrosis, which conveys a risk for underestimating the effect of NAFLD on mortality. Moreover, the prevalence of NAFLD in the population is approximately 25% [1], with a prevalence of 15% in individuals with normal BMI [56]. Thus, in studies where NAFLD patients are compared with a control population, the latter contains many individuals with NAFLD, which means that the effect of NAFLD on clinical outcomes is further underestimated. Even in strictly selected control subjects (healthy controls) in clinical trials, the risk of including NAFLD patients is substantial if liver biopsy is not used as a criterion for inclusion in either group [57]. In these circumstances, MRI is a promising non-invasive modality for defining which study subjects that are free of steatosis.

## Conclusion

During the last decade, a number of studies have shed light upon the natural history of NAFLD. There is strong evidence that patients with NAFLD have an increased risk of developing diabetes and liver-related complications [8, 36]. The highest risk of developing liver-related complications is seen in older patients with diabetes [23••, 24, 32•]. Not surprisingly, the strongest predictor of liver-related mortality is advanced fibrosis stage [22••, 23••]. It is puzzling that there is conflicting results regarding all-cause and cardiovascular mortality [27, 28], and the biggest challenge is to interpret the lack of association between NASH and liver-related mortality as well as fibrosis progression [22••, 23••, 48, 52].

Even though our knowledge on the natural history of NAFLD has increased, it is obvious that major questions remain to be answered. There are still very few longitudinal studies that evaluate clinical, biochemical, and histopathological variables that predict fibrosis progression. Hopefully, ongoing large collaborations will shed more light into the darker corners of the natural history of NAFLD.
